# Treatment of multicentric Castleman disease through combination of tocilizumab, lenalidomide and glucocorticoids: Case report

**DOI:** 10.1097/MD.0000000000017681

**Published:** 2019-11-15

**Authors:** Sisi Cai, Zhaodong Zhong, Xiang Li, Hong Xiang Wang, Li Wang, Min Zhang

**Affiliations:** a Institution of Hematology, Union Hospital, Tongji Medical College, Huazhong University of Science and Technology; b Institution of Hematology, The Central Hospital of Wuhan, Tongji Medical College, Huazhong University of Science and Technology, China.

**Keywords:** glucocorticoids target treatment, lenalidomide, unicentric Castleman's disease, tocilizumab

## Abstract

**Rationale:**

Castleman's disease (CD) is a rare lymphoproliferative disease. Compared to unicentric CD, multicentric Castleman disease (MCD) displays poorer prognosis and great variance to different therapies. Though chemotherapy, immunization therapy, and glucocorticoids have been used in the treatment of MCD, its optimal treatment is still controversial.

**Patient concerns:**

A 47-year-old woman was admitted due to poor appetite, general fatigue, puffiness of face, systemic rash, and abdominal distension. On physical examination, the patient displayed as general lymphadenopathy, splenomegaly, hepatomegaly, and shifting dullness.

**Diagnoses:**

After biopsy of her swollen lymph node and laboratory tests, her initial diagnosis was hyaline vascular-CD.

**Interventions:**

She was treated with combination of tocilizumab, lenalidomide, and glucocorticoids.

**Outcomes:**

This patient achieved complete remission (CR) with all her indexes returned to be normal. Her blood routines and biochemical examinations were still normal during the following period.

**Lessons:**

We reported a case with multicentric Castleman's disease (MCD) which acquired quite good remission after combination treatment with tocilizumab, lenalidomide, and glucocorticoids. Our report provided powerful evidence for displaying the efficiency and safety of target therapy against unicentric Castleman disease.

## 1 Introduction

Castleman disease (CD) is a rare malignant disorder characterized by lymphocytes proliferation. According to its histopathologic features, CD could be classified as hyaline-vascular, plasma-cell type and mixed-type of the former two.^[1]^ CD is also categorized as unicentric CD and multicentric Castleman disease (MCD) on the basis of affected lymph node. Unicentric CD affects single lymph node or one region of lymph nodes, while MCD involves more than 1 affected region.^[2]^

The etiology of CD is unknown, but there are studies indicated that the occurrence of CD may be related to the infection of human herpes virus-8 (HHV-8) or human immunodeficiency virus (HIV), immune dysfunction and overproduction of interleukin-6 (IL-6).^[3]^ Asao et al have proved IL-6 transgenic mice showed similar disorders related to CD, which suggested the contribution of IL-6 to CD.^[4]^

Compared with MCD, unicentric CD has a more favorable prognosis. Most patients with unicentric CD could be cured by excision of abnormal lymph node. Distinct from the former, MCD needs a systemic therapeutic conduction with poorer prognosis. In addition, MCD displays great variance to different therapies. So far, though chemotherapy, immunization therapy, and glucocorticoids have been used in the treatment of MCD, its optimal treatment is still controversial.^[5]^

Here, we report a newly-diagnosed case of MCD without the detection of HHV and/or HIV infection. This patient displayed very sick when she was transferred from a local hospital with a series of clinical symptoms and signs such as weakness, magersucht, fever, ascites syndrome, and so on. This case received the treatment regimen including glucocorticoids, lenalidomide and anti-interleukin-6 receptor antibody (Tocilizumab).

## 2 Case report

A 47-year-old female patient was admitted to local hospital for poor appetite, general fatigue, puffiness of face, systemic rash, and abdominal distension in May 2016. Her physical examination displayed as general lymphadenopathy, splenomegaly, hepatomegaly, and shifting dullness. Through biopsy of her swollen lymph node, hyaline vascular-CD was identified, with the immunohistochemistry results of CD20 positive, CD30 positive, CD138 negative, and CD38 positive. Her ascites were canary, slight turbidity, of which chloridion was 109.3 mmol/L, glucose was 6.55 mmol/L, total protein was 31.0 g/L, LDH was 197 U/L. Occult blood and protein were detected in her urine, the concentrations of white blood cell and red blood cell in her urine was 23/μL or 7/μL, respectively. Autoimmune diseases were excluded for undetected evidences. Positron emission tomography/computer tomography (PET/CT) in local hospital revealed that multiple swollen lymph nodes in different sizes were detected in bilateral axillary fossa. In addition, bilateral pleural effusions, massive pericardial effusion, ascites, pelvic effusion, and splenomegaly were found through PET-CT scan.

This patient was transferred to our hospital for further examination and treatment on May 2016. Her blood routine analysis showed that white blood cell count was 6.56 × 10^9^/L, Hemoglobin was 12.6 g/dL, platelet number was 265 × 10^9^/L. Some inflammatory indexes, such as erythrocyte sedimentation rate (ESR) (65 mm/hour) and C reaction protein (CRP) (25.2 mg/L) were obviously increase compared to normal control. Her serum albumin and gama-globulin values were 30.0 g/L and 21.30 g/L individually, her serum creatinine level was 192.9 μmol/L and blood urea nitrogen was 17.37mmol/L. The bone marrow (BM) morphology for this patient displayed as normal with the normal karyotype of 46, XX [20]. Computed tomography (CT) scan indicated pneumonia, hydrothorax, pericardial effusion, ascites, splenomegaly. Numerous swollen lymph nodes were detected in bilateral axillary fossa, inguinal grooves, mediastinum, and retroperitoneum. The results of echocardiography and electrocardiograph displayed as followed: enlargement of right heart, moderate insufficiency of 3 apical valve, widening of pulmonary artery valve and decreased diastolic function of left ventricle of pulmonary artery, medium to large pericardial effusion. Serum IL-6 level was 157.86 pg/ml at first. Tests for human immunodeficiency virus (HIV) and human herpesvirus 8 (HHV-8) were negative.

**Figure 1:**
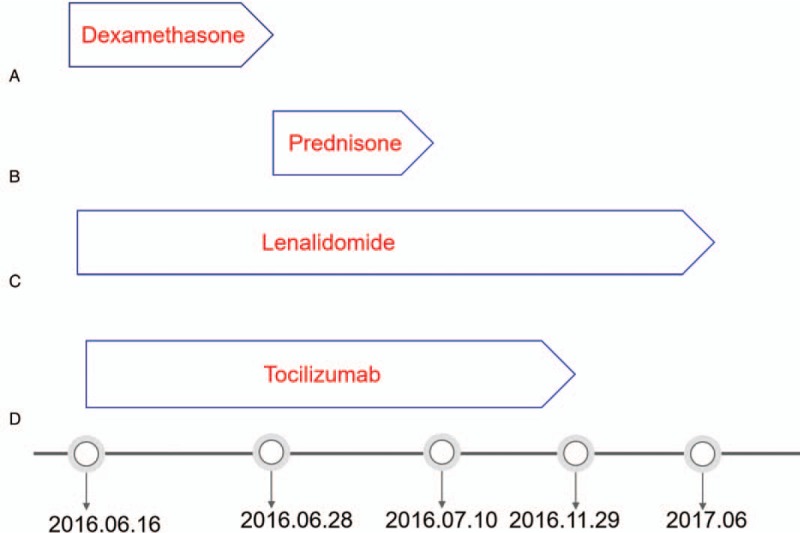
A. Dexamethasone was initiated intravenous at a dose of 7.5 mg per day first from 16th June 2016 to 28th June 2016. B. Oral prednisone treatment (40 mg daily) was initiated with the reduction of its dose by half every 3 days from 16th June 2016 to 10th July 2016. C.25 mg of Lenalidomide was administrated on days 1 to 21 in a 28-day cycle from 16th June 2016 and suspended on June 2017. D. Tocilizumab was given at a dose of 8 mg/kg every 2 weeks from 16th June 2016 to 29th November 2016.

The treatment in our hospital was initiated from June 2016. Because of her poor health condition, this patient was administrated dexamethasone(Zhejiang Xianju Pharmaceutical Co., Ltd) intravenous at a dose of 7.5 mg per day first for 13 days, and then prednisone (Zhejiang Xianju Pharmaceutical Co., Ltd) treatment (40 mg daily) was initiated for another 70 days, the dose of which was reduced by half every 3 days until drug withdraw. Based on the experience with multiple myeloma and according to Mayer J’ report, 25 mg of Lenalidomide (Celgene International Sarl company) was administrated on days 1 to 21 in a 28-day cycle, it was suspended until June, 2017, after 11 cycles was completed. 8 doses of tocilizumab (Roche Holding AG) were given at a dose of 8 mg/kg every 2 to 4 weeks from 16th June to 29th November 2016 (Fig. 1). During the treatment period, we dynamically monitored the biochemical indicators and the blood Routine of the patients and probiotics and diuretics were administrated to patient for controlling watery diarrhea and ascites. The patient was well tolerated with the treatment.

**Figure 2:**
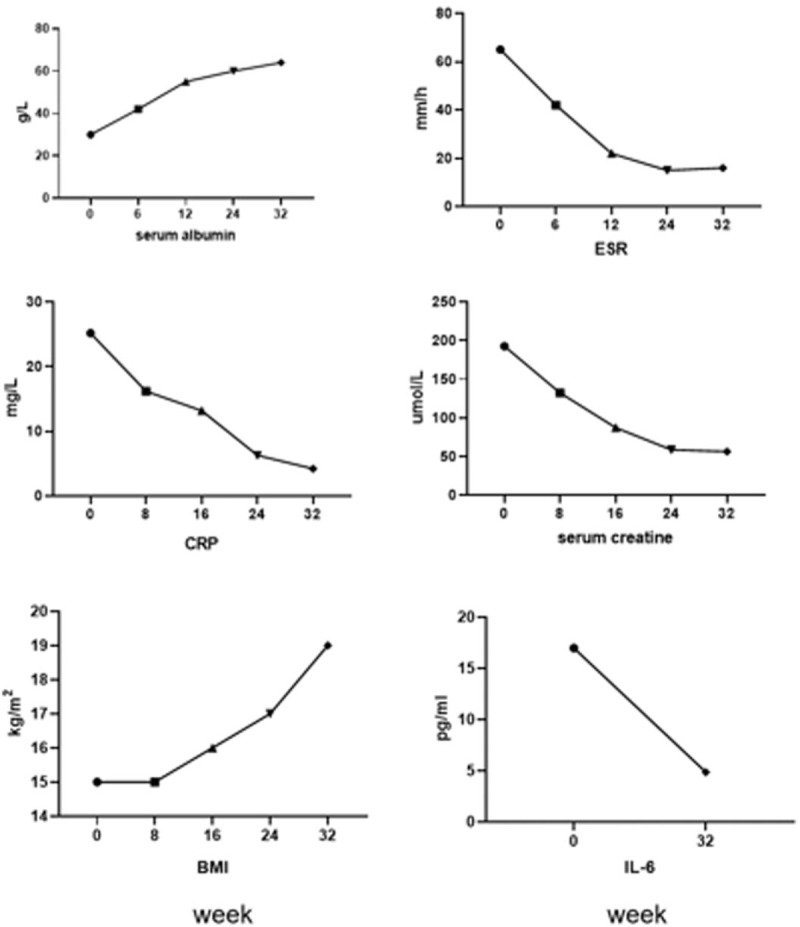
In the course of treatment, we monitored the patients’ indexes of biochemistry and inflammatory dynamically, they achieved complete remission with all her indexes returned to be normal within 32 weeks.

**Figure 3:**
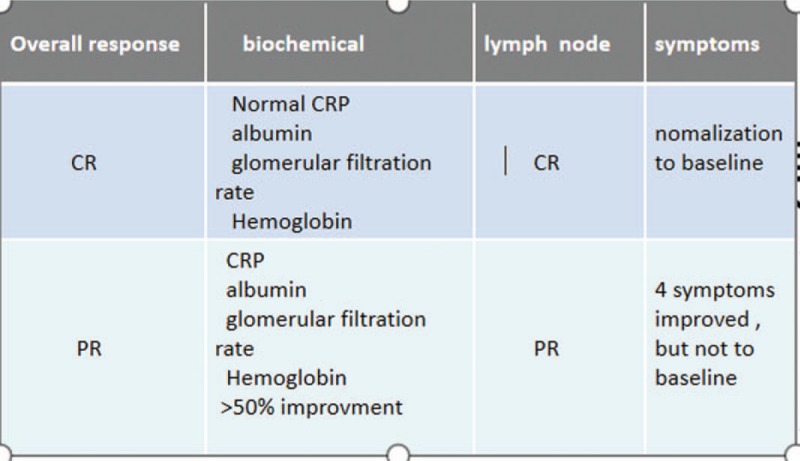
Castleman Disease Collaborative Network response criteria was based on the evaluation of biochemical indexes and lymphadenopathy an overall CR requires biochemical, lymph nodes, and symptomatic responses. Symptoms include fatigue, fever, weight loss, and anorexia.

The patient acquired a favorable treatment effect: the shrink of superficial lymph nodes around the whole body, normalized sizes of liver and spleen, increased appetite and extinction of edema, etc. The symptoms and biochemical abnormities were improved after combination treatment with tocilizumab, lenalidomide, and glucocorticoids over 2 months. In addition, PET-CT result after treatment showed that both the numbers and the metabolic activity of superficial and retroperitoneal lymph nodes were decreased significantly compared to the data in May 2016. And then, this patient achieved complete remission (CR) with all her indexes returned to be normal since December, 2016. (Figs. 2 and 3) In addition, this patients’ weight was returned to the level before her illness, she resumed her job, her blood routines and biochemical examinations were still normal until May 2019.

## 3 Discussion

As above mentioned, It was reported that chemotherapy, especially CHOP (cyclophosphamide, doxorubicin, vincristine, and prednisone) regimen, or combined Rituximab (an anti-CD 20 monoclonal antibody) with CHOP regimen, or even single use of Rituximab, are widely-used for treatment of CD.^[1]^ Linus et al suggested that Rituximab combination with corticosteroids should be considered, especially in severe cases.^[6]^ Also, Rituximab combination with CHOP regimens has been used in patient with CD based on lymphoma treatment.^[6–8]^

Lenalidomide is an analogs and derivative of thalidomide, has showed immunomodulatory and anti-angiogenic effects, could decrease the production of IL-6, too. Its application in CD controlled the patients’ condition well with good safety.^[1–2]^ The most common side effects of lenalidomide were thrombocytopenia (61.5%) and neutropenia (58.8%). Other common side effects include diarrhea, itching, rash, fatigue, and constipation. But we can prevent and ameliorate it by using granulocyte colony-stimulating factors.^[6–7]^ During the therapy time, only mild leucopenia was appeared and improved by subcutaneous injection of granulocyte colony-stimulating factor (Qilu Pharmaceutical Co, Ltd).

IL-6 is a cytokine that stimulates the growth and differentiation of B-lymphocytes and is also a growth factor for hybridomas and plasmacytomas.^[3–6]^ Previous studies showed that anti-IL-6 antibody or anti-IL-6 receptor antibody (tocilizumab) dramatically alleviated symptoms and biochemical abnormalities of MCD.^[3–6,10,11]^ Combination therapy of glucocorticoids and tocilizumab can be effective for CD with thrombocytopenia, anasarca, myelofibrosis, renal dysfunction, and organomegaly (TAFRO) syndrome.^[7,12]^ HHV-8 and/or HIV infections were detected in most of patients with MCD, while some HHV-8-negative and/or HIV-negative patients were also existed and called as idiopathic multicentric Castleman disease (iMCD).^[1]^ This case should belong to iMCD due to un-detection of HIV and/or HHV-8. Although anti-IL6 monoclonal antibody (siltuximab) is the only treatment for iMCD approved by the United States of America Food and Drug Administration (FDA), it is not universally effective.^[8]^ A phase II clinical trial including 79 subjects shows that iMCD patients exhibiting a clear constellation of abnormal inflammatory parameters, including CRP, fibrinogen, IgG were most appropriate for siltuximab therapy.^[8–9]^

Tocilizumab, one kind of interleukin-6 receptor antibodies was approved for MCD therapy by Japanese FDA in 2005.^[13–15]^ An international Working Group comprising 42 experts from 10 countries was convened by the Castleman Disease Collaborative Network to establish iMCD consensus management guidelines, The working group reviewed data from 344 cases, published literature, and referred to expert opinion. The group advised that the anti–interleukin-6 monoclonal antibody tocilizumab with or without corticosteroids can be the preferred substitutes if siltuximab is not available.^[16]^ In China, although tocilizumab has not been approved for MCD therapy by FDA, it is affordable and the only obtained antibody against iMCD here, after enough communication with this patient, adequate treatments were initiated with strict monitoring. To obtain the optimal treatment effects, combination of tocilizumab with lenalidomide and glucocorticoid were administrated for this patient.^[10–12]^

In this report, the patient showed cytopenia, fatigue, anorexia, weakness, fever, pneumonia, severe multi-cavity effusion, renal function failure. Combination of glucocorticoids with lenalidomide and tocilizumab for more than 2 months was used on the patient. PET-CT scan demonstrated the decrease of lymphadenopathy and splenomegaly after treatment. In addition, cavity effusion, renal insufficiency was corrected with the biochemical abnormities alleviated. The patient obtained the freedom of movement.

Until May 2019, this patient has acquired and maintained CR for over 20 months without any discomfort. Our treatment regimen including tocilizumab combination with lenalidomide and glucocorticoids has shown significant efficacy on MCD without obvious side effects. This target therapy regimen might be alternative treatment for CD after replacement of chemotherapy in the future, if much more clinical data were achieved and more cases were involved.

## Author contributions

**Conceptualization:** Min Zhang.

**Data curation:** Xiang Li, Hongxiang Wang, Li Wang.

**Investigation:** Hongxiang Wang.

**Supervision:** Min Zhang.

**Validation:** Hongxiang Wang.

**Writing – original draft:** Sisi Cai, Zhaodong Zhong.

**Writing – review & editing:** Min Zhang.
